# Application of Machine Learning for Cytometry Data

**DOI:** 10.3389/fimmu.2021.787574

**Published:** 2022-01-03

**Authors:** Zicheng Hu, Sanchita Bhattacharya, Atul J. Butte

**Affiliations:** ^1^ Bakar Computational Health Sciences Institute, University of California, San Francisco, San Francisco, CA, United States; ^2^ Department of Microbiology and Immunology, University of California, San Francisco, San Francisco, CA, United States

**Keywords:** cytometry, cyTOF, machine learning, predictive modeling, flow cytometry

## Abstract

Modern cytometry technologies present opportunities to profile the immune system at a single-cell resolution with more than 50 protein markers, and have been widely used in both research and clinical settings. The number of publicly available cytometry datasets is growing. However, the analysis of cytometry data remains a bottleneck due to its high dimensionality, large cell numbers, and heterogeneity between datasets. Machine learning techniques are well suited to analyze complex cytometry data and have been used in multiple facets of cytometry data analysis, including dimensionality reduction, cell population identification, and sample classification. Here, we review the existing machine learning applications for analyzing cytometry data and highlight the importance of publicly available cytometry data that enable researchers to develop and validate machine learning methods.

## Introduction

Flow cytometry has been widely used in both research and clinical settings to characterize biological samples at single-cell resolution with multiple protein markers. Researchers first label the cells with fluorescent-tagged antibodies and use a flow cytometer to detect the fluorescent signals as the cells rapidly flow past lasers. Since its first use in the 1960s (1, 2), the basic design of flow cytometry remains largely unchanged. However, continuous improvements have been made to the flow cytometers and fluorescent dyes, significantly increasing the speed at which cells are analyzed and the number of protein markers that can be detected. Cytometry by time of flight (CyTOF, as known as mass spectrometry) was invented in the 2000s (3, 4). Through the use of heavy metal isotope-coupled antibodies, CyTOF can detect isotope peaks without significant spectrum overlap, thus profiling more than 50 protein markers simultaneously.

While other advanced technologies, such as single-cell RNA-sequencing (scRNA-seq) and Cellular Indexing of Transcriptomes and Epitopes by Sequencing (CEIT-seq), offer to characterize the cells with a much larger number of measurements, their use is limited by the high cost and the relatively low number of cells that can be processed. Conversely, the low cost of the cytometry experiment allows it to be used to characterize hundreds of samples, while most scRNA-seq experiments are limited to less than 10 samples. The cytometry is also capable of profiling a large number of cells (> 10^6) per sample, allowing researchers to identify rare cell populations that could potentially be missed by scRNA-seq. Thus, modern cytometry remains to be one of the most important tools for immunology research.

The analysis of cytometry data remains to be a challenge due to its high dimensionality and the large number of cells. The traditional manual gating uses a series of two-dimensional plots to visualize the data and uses hierarchical gates to identify cell populations. A key advantage of manual gating is that it allows researchers to incorporate existing knowledge into the cytometry data analysis, including the function of protein markers and the developmental relationship of the cell populations. However, it faces significant challenges when analyzing high-dimensional cytometry data, as the two-dimensional plots often fail to show the complex high-dimensional structure of the data. Moreover, there is a possibility of human bias while analyzing data from manual gating. In clinical settings, manual gating also suffers from additional disadvantages such as the low processing speed and the susceptibility to human errors.

To overcome the challenges faced by manual gating, many computational tools have been developed to automate every step of the cytometry data analysis, including quality control (5), batch normalization (6, 7), data visualization (8–10), cell population identification (11–16), and sample classification (17–20). The tools utilize a wide range of computations methods, ranging from rule-based algorithms to machine learning models. Machine learning is a set of computational and statistical methods that learn patterns from the data with minimal input from humans. The machine learning methods can be classified as supervised and unsupervised learning (21) depending on if external labels, annotation or prior information are available. In cytometry analysis, machine learning models have been primarily used for dimensionality reduction, cell population identification, and sample prediction (11–20). In this review, we discuss different machine learning approaches for analyzing flow cytometry data and the challenges faced by these approaches. We also highlight the importance of publicly available cytometry data that enabled researchers to develop machine learning methods.

## Machine Learning Methods for Dimensionality Reduction

Data visualization is often the first step in data analysis and can have a profound influence on the subsequent interpretation of high-dimensional cytometry data. By representing the high dimensional data in two or three-dimensional graphs, it enables researchers to explore the data and recognize patterns that can be tested by later statistical analysis. In addition, data visualization graphics are frequently displayed in publications to convey biological insights (22, 23). Therefore, it is necessary to ensure that the low dimensional visualization accurately represents the information in the original data. The toolbox for dimensionality reduction is expanding rapidly. Researchers now have a wide variety of methods at their disposal for data visualization, including Principle Component Analysis (PCA), t-Distributed Stochastic Neighbor Embedding (tSNE), UMAP, and many more (9, 24–26). Information loss is almost inevitable when high-dimensional data is compressed into two or three dimensions for visualization. Different dimensionality reduction methods are designed to preserve various aspects of information in data. Methods such as PCA and Multidimensional scaling (MDS) aim to best preserve the global structure in the data (27); Embedding methods such as tSNE and UMAP aim to preserve the local structure in the data (25, 28). tSNE and UMAP are particularly suitable for visualizing cytometry data due to their ability to separate major cell subsets when projecting the high dimensional data into two-dimension. However, cautions need to be taken when interpreting the specific aspect of the tSNE and UMAP plots. The distances between the cells are often distorted in tSNE and UMPA plots. Thus, the similarity between cells should be assessed using distance measures based on the original high-dimensional space. Cell clusters should also be identified using the original data rather than the low-dimensional data from tSNE and UMAP.

## Machine Learning Methods for Cell Population Identification

Researchers routinely use cytometry to profile the cell populations in biological samples. Data from cytometry experiments not only allows researchers to understand the cellular composition of healthy tissues but also provides valuable information about how different cell subsets change in disease conditions (4, 29–31). Many machine-learning methods have been developed to annotate established cell populations, as well as to discover novel cell subsets from the high-dimensional cytometry data (**Table 1**).

**Table 1:** Selected machine learning methods for cytometry analysis.

Machine learning type	Name	Desciption
Dimentionality reduction	PCA	PCA projects the high-dimensional data into lower dimensions while preserving as much of the data's variation as possible.
	MDS	MDS projects the high-dimensional data into lower dimensions while preserving as much of the pairwise distances between the cells. MDS and PCA are equivalent when the Euclidean distance is used.
	tSNE	t-SNE (t-distributed stochastic neighbor embedding) is a non-linear dimensionality reduction method. t-SNE transforms the pairwise distances into probabilities based on t-distribution, thus emphasizing preserving the data's local structure.
	UMAP	UMAP (Uniform Manifold Approximation and Projection) is a method for dimension reduction using manifold learning techniques. Similar to tSNE, UMAP emphasis preserving the local structure of the data.
Unsupervised methods for cell population identification	FLOCK	FLOCK identify cell populations using density-based clustering.
	flowSOM	FlowSOM maps cells to self-organizing maps and uses consensus hierarchical clustering to identify the cell populations.
	flowMeans	flowMeans uses K-means clustering a change point detection algorithm to identify cell populations.
	flowMerge	FlowMerge first uses Gaussian mixture models to identify cell subsets from the cytometry data and uses entropy-based criteria to merge the closely related cell population.
	MetaCyto	MetaCyto uses a combination of hierarchical clustering and cell population labeling to identify shared cell populations across studies.
	SWIFT	Swift uses a Gaussian mixture model-based clustering method to identify cell subsets, followed by splitting and merging steps to adjust the number of clusters to identify rare subpopulations
	PhenoGraph	PhenoGraph first constructs a nearest neighbor graph of the single cells based on their phenotypic similarity and then partition the graph into clusters using a community detection algorithm.
Supervised methods for cell population identification	LDA for cytometry data	The method train a linear discriminant analysis (LDA) classifier to identify cell populations
	DGCyTOF	DGCyTOF trains a deep learning model to identify cell populations. A feedback loop is included to adjust between new and unknown cell populations.
	DeepCyTOF	DeepCyTOF trains a deep learning model to identify cell populations. DeepCyTOF includes a calibration step to adjust for batch effects between datasets.
Sample classification using cell subset information	CITRUS	CITRUS uses hierarchical clustering to identify a large number of small cell subsets from cytometry data and uses a LASSO model to predict clinical outcomes.
	FloReMi	FloReMi is a pipeline for data preprocessing, cell subset identification, feature selection, and predictive modeling of cytometry data. FloReMi uses a Random Forest model to predict clinical outcomes using cell subset information.
Sample classification using single-cell data	CellCNN	CellCNN adopted a convolutional neural network structure to predict clinical or biological outcomes directly using single-cell data from cytometry experiments.
	Deep CNN	The Deep CNN model uses a convolutional neural network structure to predict clinical or biological outcomes directly using single-cell data from cytometry experiments. The model includes a higher number of internal layers, allowing the model to better capture the complex interactions between cell marks in the cytometry data.

### Unsupervised Machine Learning Methods for Cell-Type Identification

**Figure 1:**
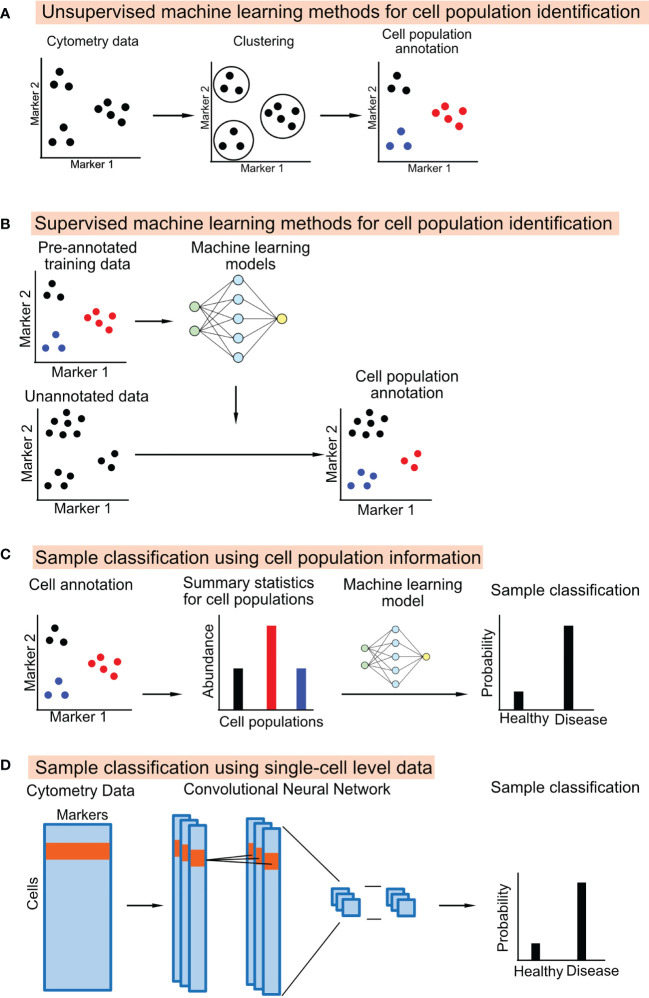
Schematic diagrams showing the machine learning approaches used to annotate cell population from cytometry data or classifying the cytometry samples.

Unsupervised machine learning methods identify groups of cells that are similar to each other based on cytometry data itself without external information (**Figure 1A**). Many generic un-supervised methods can be applied directly to cytometry data, including the popular clustering methods such as K-means clustering and hierarchical clustering, the probability-based methods such as gaussian mixture models, and density-based methods such as HDBSCAN (32).

Researchers have also developed computational pipelines that are optimized for cytometry data, including FLOCK, flowSOM, flowMeans, flowMerge, SWIFT, PhenoGraph, and many other methods (11–16, 33). These pipelines use a combination of existing unsupervised machine learning methods and customized algorithms to optimize the analysis workflow. For example, flowSOM maps the cells to a self-organized map (SOM) and performs consensus hierarchical clustering to identify the cell populations (15). FLOCK first identify regions with high densities of cells and later merges the adjacent high-density regions into cell populations (16). Unlike many other unsupervised methods, FLOCK does not require users to pre-define the number of cell populations, although additional hyperparameters still need to be tuned by the user to optimize the results. FlowMerge uses Gaussian mixture models to identify cell subsets from the cytometry data (13). To address the problem that the mixture models often overestimate the number of cell populations, flowMerge uses entropy-based criteria to merge the closely related cell population. The PhenoGraph first constructs a nearest neighbor graph of the single cells based on their phenotypic similarity and then partition the graph into clusters using an efficient community detection algorithm (33, 34).

There are advantages of applying unsupervised methods to enumerate cell populations in high-dimensional space in an unbiased fashion, which is not possible using the manual gating approaches. The methods also make it possible to automate the identification of cell populations with minimal input from humans. At the same time, the unsupervised methods face multiple challenges. First, identified cell populations are computed without any prior knowledge and are not directly interpretable. Researchers often need to manually inspect the expression of different markers to determine the cell population identity. Second, many unsupervised methods tend to ignore rare cell populations. A potential solution is to conduct multiple rounds of clustering to identify small cell subsets within the major cell populations. Finally, most unsupervised methods can only be applied to data from a single experiment. Cell populations identified from different datasets are often not directly comparable with each other. If possible, researchers should try to combine the datasets using batch correction methods, such as cytoNorm (7), before applying the unsupervised machine-learning methods. Alternatively, researchers could use adaptive methods, such as MetaCyto (11), to identify the same set of cell populations from different datasets while taking the batch effects from each dataset before merging multiple datasets from different sources.

### Supervised Machine Learning Methods for Cell-Type Identification

A supervised machine learning method learns a classifier from training datasets, which consists of cytometry data and the manually annotated cell-type information. The learned classifier can then be applied to annotate new cytometry datasets (**Figure 1B**). One study uses a linear discriminant analysis (LDA) classifier to annotate the cell types in CyTOF data (35). Other studies used neural network models for cell annotation, including DGCyTOF and DeepCyTOF. DGCyTOF designed a customized neural network model to adjust between new and unknown cell populations *via* a feedback loop, which reduces the rate of error in the identification of cell types (36). The DeepCyTOF includes a calibration step to adjust for batch effects between datasets, allowing the trained model to be applied to multiple datasets (37).

While compared to unsupervised methods, the supervised methods allow researchers to guide the cell type annotation by providing training labels. In addition, it is possible to train the supervised models using heterogeneous datasets, thus improving the generalizability of the models. On the other hand, the quality of the supervised models depends on the human-provided labels and can potentially mirror the human bias. The supervised models can only identify known cell types that have been annotated by humans. Therefore, a combination of supervised and unsupervised methods should be used to identify known cell populations, as well as to discover novel cell subsets.

## Machine Learning Methods for Sample Classification

Cytometry is widely used to identify biomarkers that can be used for disease diagnosis or prognosis. Previous studies have reported the use of cytometry in diagnosing multiple types of diseases, including leukemia, allergies, and infectious diseases (38–40). Cytometry can also be used to predict other types of clinical outcomes, such as the response to vaccination and to cancer immune-therapies (41, 42). Multiple machine learning methods have been developed to predict clinical or biological outcomes by classifying the cytometry sample into groups (**Table 1**), such as health vs. disease, responders vs non-responders, and more. Similar approaches can also be used to predict continuous outcomes using regress models.

### Sample Classification Using Cell Subset Information

In most studies, biomarker discovery and predictive modeling are downstream steps to the cell-type annotation step in the cytometry data analysis pipeline. The summary statistics of the cell populations, including their abundance and their median or mean marker intensities, can be used as features to classify the cytometry samples (**Figure 1C**). Many studies directly apply machine learning models to the cell population information, such as logistic regression, random forest, and gradient boosted trees (43–46). Pipelines and applications have also been developed for cytometry data. E.g., CITRUS applies an unsupervised hierarchical clustering method to identify a large number of small cell subsets. CITRUS then uses the Least Absolute Shrinkage and Selection Operator (LASSO) model to predict outcomes of interest from the cell subset information (17). The L1-regularization of the LASSO model allows researchers to identify the most informative cell subset information for prediction. FloReMi is a pipeline developed from the FlowCAP IV challenge to predict the time until progression to AIDS for HIV patients using cytometry data but could be easily adapted for other prediction problems (18). The pipeline contains multiple steps for predictive modeling using cytometry data, including data preprocessing, cell subset identification, feature selection, and predictive modeling.

Predictive modeling using cell subset information is highly intuitive and interpretable. Researchers can easily identify the most predictive cell types associated with the outcome of interest. The approach is straightforward to implement. Generic machine learning models can be directly applied to the cell subset information. However, the approach faces several challenges. First, the cell-subset identification step is disconnected from the latter predictive modeling step. Therefore, the identified cell subsets are often not optimized for identifying cell populations that are most associated with the outcome of interest. For example, the cell type identification process may miss rare cell populations that are key to disease prognosis. Second, the original cytometry data are reduced to summary statistics of cell subsets, potentially leading to the loss of important information such as the correlation between cell markers and the distribution of marker expression within each cell subset. Third, the approach requires all samples to be clustered in the same way, making it sensitive to batch effects and the choice of clustering methods. Finally, the approach may fail to detect cellular changes that do not lead to distinct cell populations, such as the continuous up-regulation of CTLA-4 in T cells in response to varying degrees of stimulation.

### Predictive Modeling Using Single-Cell Data

Several studies have used a different approach for predictive modeling. Instead of using cell subset information, machine learning models can be directly applied to the single-cell level cytometry data to predict outcomes (**Figure 1D**). The input of the model is the protein marker profiles of randomly ordered cells from a cytometry sample; the output of the model is the clinical or biological outcome associated with the cytometry sample.

Most commonly used supervised models require the input to be a single vector of features, such as logistic regression, random forest, and gradient boosted trees. Because cytometry data is a collection of randomly ordered single-cell profiles, it is challenging to build predictive models using these supervised learning methods. Researchers thus turn to neural network models, which have been proven to be highly flexible for handling a wide range of structured and unstructured data as inputs.

CellCNN is the first neural network designed to predict outcomes using single-cell level data (19). CellCNN adopted a convolutional neural network structure and used a set of filters to extract information from the single cells. The cell-level information is summarized into sample-level information by taking the mean or maximum across all cells. The sample level information is then associated with outcomes using dense neural network layers. Another study designed a similar convolutional neural network model with a larger number of internal layers, allowing the model to better capture the complex interactions between cell marks in the cytometry data (20).

This approach directly uses the single-cell level cytometry data, circumventing the cell-type identification step. Thus, the approach avoids information loss in the cell gating step. The neural network models can be directly applied to raw cytometry data and predict outcomes in an end-to-end fashion, making it easy to optimize the prediction pipeline globally. In addition, it is possible to train the supervised models using heterogeneous datasets, thus improving the generalizability of the models. On the other hand, this approach faces several challenges. First, the approach is computationally expensive, as it uses single-cell level data and deep neural networks. Second, the models are less intuitive and do not directly allow researchers to identify cell types that are associated with clinical outcomes. Flow-up analysis needs to be conducted to interpret the model and identify cell subsets as biomarkers.

## Resources for Developing Machine Learning Models

Publically available cytometry data are valuable resources for developing, validating, and evaluating machine learning models for cytometry data analysis. Most of the machine learning tools mentioned above have either been developed using publicly available datasets or applied to public datasets for validation and evaluation. Researchers are able to improve the generalizability of the methods by testing the machine learning models using heterogenous cytometry datasets.

ImmPort is a data repository for sharing clinical and basic research data from immunology-related studies (47). As of Sep 2021, 495 studies are shared on Immport. Among them, 185 studies contain flow cytometry data or CyTOF data. As one of the oldest available immunological databases, ImmPort shares a variety of data types, including clinical data, protocols, sequencing data, cytokine profiles, antibody titers, and many more, to thousands of researchers every year. These rich datasets provide a valuable opportunity for researchers to develop and test machine learning models that are capable of predicting clinical or biological outcomes using cytometry data. In addition, ImmPort shares the cell-gating results provided by the authors of the datasets, allowing researchers to benchmark the performance of cell-population identification methods by comparing the results with manual-gating results.

FLowRepository is a database specifically designed for sharing cytometry data (48). As of Sep 2021, FLowRepository contains 1375 cytometry datasets and their associated metadata. FlowRepository evaluates the datasets based on the Minimum Information about a Flow Cytometry Experiment (MIFlowCyt) standard (49) and assigns a MIFlowCyt score for each dataset, allowing researchers to select a cytometry dataset based on the completeness of metadata. FlowRespository also hosts several datasets used by the FlowCAP challenges, which were established to compare the performance of computational methods on cell population identification and sample classifications (50). As the performance of many existing methods has been assessed using the FlowCAP datasets, researchers can use the FlowCAP datasets to benchmark the performance of new machine learning methods against the existing methods.

While a large number of cytometry datasets are publicly available, several challenges exist for researchers to apply machine learning methods to these datasets. First, the metadata of the datasets is not standardized, including the use of non-standardized names for protein markers, sample types, experimental conditions, and disease states. Data harmonization and standardization efforts are needed to unify the metadata across studies. Second, the cytometry data from different studies are highly heterogeneous, with differences in antibody panels, fluorophore combinations, cytometer instruments, and sample processing protocols. Thus, novel machine learning techniques are needed to make the models robust to these heterogeneities. Finally, only a small percentage of cytometry datasets are shared publicly. There are still only a few thousand cytometry datasets being publicly available, a number that is much smaller than the shared number of transcriptomics data (160010 transcriptomics datasets are available on GEO as of Sep 12, 2021). This is partially due to the fact that journals and funding agencies do not mandate the sharing of cytometry data, and partially due to the community’s lack of enthusiasm in repurposing the shared cytometry data. Thus, all shareholders, including researchers, journals, funding agencies, and private companies, should work together to promote the availability and utility of publicly available cytometry data.

## Concluding Remarks and Future Directions

Many machine learning-based methods have been developed for analyzing cytometry data. The machine learning models have been primarily used to annotate cell populations and to classify the cytometry samples. Early studies have used relatively simple machine learning models to automate specific steps in the cytometry data analysis pipeline while several recent studies have started to implement complex deep learning models to perform predictive modeling in an end-to-end fashion. While existing machine learning models allow researchers to analyze cytometry data with greater accuracy and speed, many challenges remain to be solved. First, most machine learning models were designed to analyze data from a single study. More robust machine learning models are needed to enable the analysis of heterogeneous datasets. Second, the current machine learning models fail to incorporate existing biological knowledge into the cytometry analysis. Novel machine learning models, such as transfer learning models, could potentially be used to improve cytometry data analysis. Finally, the results from many machine learning methods are difficult to interpret. New model interpretation methods are needed to allow researchers to understand the machine learning results and to extract biological insights from the model. At the same time, the whole community should work together to promote the availability and standardization of publicly available cytometry data, providing richer resources for developing new machine learning models.

## Author Contributions

ZH formulated the original idea and reviewed the manuscript. SB contributed to the design of the review. SB and AB reviewed the manuscript. All authors contributed to the article and approved the submitted version.

## Funding

This work was supported by the National Institute of Allergy and Infectious Diseases ImmPort contract HHSN316201200036W (to AB) and research grant UH2 AI153016 (to ZH).

## Author Disclaimer

The content is solely the responsibility of the authors and does not necessarily represent the official views of the National Institutes of Health.

## Conflict of Interest

AB is a co-founder and consultant to Personalis and NuMedii; consultant to Samsung, Mango Tree Corporation, and in the recent past, 10x Genomics, Helix, Pathway Genomics, and Verinata (Illumina); has served on paid advisory panels or boards for Geisinger Health, Regenstrief Institute, Gerson Lehman Group, AlphaSights, Covance, Novartis, Genentech, and Merck, and Roche; is a shareholder in Personalis and NuMedii; is a minor shareholder in Apple, Facebook, Google, Microsoft, Sarepta, 10x Genomics, Amazon, Biogen, CVS, Illumina, Snap, Nuna Health, Assay Depot, Vet24seven, Regeneron, Moderna, and Sutro, and several other non-health related companies and mutual funds; and has received honoraria and travel reimbursement for invited talks from Genentech, Takeda, Varian, Roche, Pfizer, Merck, Lilly, Mars, Siemens, Optum, Abbott, Celgene, AstraZeneca, AbbVie, Johnson and Johnson, Westat, and many academic institutions, state or national agencies, medical or disease specific foundations and associations, and health systems. AB receives royalty payments through Stanford University, for several patents and other disclosures licensed to NuMedii and Personalis. AB’s research has been funded by NIH, Robert Wood Johnson Foundation, Peraton (formally known as Northrop Grumman) as the prime on an NIH contract, Genentech, Johnson and Johnson, FDA, the Leon Lowenstein Foundation, the Intervalien Foundation, Priscilla Chan and Mark Zuckerberg, the Barbara and Gerson Bakar Foundation, and in the recent past, the March of Dimes, Juvenile Diabetes Research Foundation, California Governor’s Office of Planning and Research, California Institute for Regenerative Medicine, L’Oreal, and Progenity. SB and ZH are funded by ImmPort (under UCSF subcontract with Peraton). ZH is the author of MetaCyto and deep CNN for cytometry data analysis.

## Publisher’s Note

All claims expressed in this article are solely those of the authors and do not necessarily represent those of their affiliated organizations, or those of the publisher, the editors and the reviewers. Any product that may be evaluated in this article, or claim that may be made by its manufacturer, is not guaranteed or endorsed by the publisher.
